# Histomorphologic characteristics of abdominal aortic aneurysm samples are similar in abdominal-only vs thoracoabdominal involvement

**DOI:** 10.1016/j.jvssci.2026.100424

**Published:** 2026-05-08

**Authors:** Marvin Kapalla, Heiner Nebelung, Anna Klimova, Anja Hofmann, Nadja Sachs, Albert Busch, Maja Carina Nackenhorst

**Affiliations:** a Division of Vascular and Endovascular Surgery, Department for Visceral, Thoracic and Vascular Surgery, Medical Faculty Carl Gustav Carus and University Hospital, Technische Universität Dresden, Dresden, Germany; b Institute and Polyclinic for Diagnostic and Interventional Radiology, Faculty of Medicine and University Hospital Carl Gustav Carus, Technische Universität Dresden, Dresden, Germany; c Institute for Medical Informatics and Biometry, Medical Faculty and University Hospital Carl Gustav Carus, Technische Universität Dresden, Dresden, Germany; d Department for Vascular and Endovascular Surgery, Klinikum rechts der Isar, Technical University Munich, Munich, Germany; e German Center for Cardiovascular Research (DZHK), partner site Munich Heart Alliance, Berlin, Germany; f Institute of Molecular Vascular Medicine, TUM Klinikum, Technische Universität München (TUM), Munich, Germany; g Department of Pathology, Medical University of Vienna, Vienna, Austria

**Keywords:** Abdominal aortic aneurysm, Fibrosis, Histomorphology, Inflammation, Thoracoabdominal aortic aneurysm

## Abstract

The abdominal aorta is the most frequent site of aneurysm development including mostly localized abdominal aortic aneurysms (AAAs) but also more extensive pathologies like thoracoabdominal aneurysms (TAAAs). This study compares histologic features including inflammation, fibrosis, and angiogenesis in samples from the abdominal aneurysm wall from patients with AAAs (n = 272) and TAAAs (n = 24) after open repair. Both cohorts show a similar pathomorphology and a patient-centered propensity score–matching for age, sex, clinical presentation, maximum diameter, comorbidities, smoking, and basic medication reveals no significant differences for the basic histologic features investigated. These exploratory results can be interpreted towards a possible transferability of basic and translational research results for a nonsurgical treatment of patients with AAAs and TAAAs.

**Clinical Relevance:**

Abdominal aortic aneurysm is by far the most frequent aortic aneurysm—yet basic scientific knowledge is limited, and clinical studies on aneurysm growth abrogation have not shown beneficial effects. Data and understanding on more extensive disease, such as thoracoabdominal aortic aneurysms is even more scarce. This manuscript provides a first hint that pathomechanisms based on histomorphology might be comparable between abdominal aortic aneurysm samples and abdominal wall samples from patients with thoracoabdominal aortic aneurysms in a well-matched propensity-scored analysis. Thus, results from translational research on aneurysm growth alterations could be applicable for both entities.

**Article Highlights**

• **Type of Research:** Experimental cohort study
• **Key Findings:** We conducted histologic evaluation of 272 abdominal aortic aneurysm samples and 24 abdominal aortic wall thoraco-abdominal aneurysm samples. Patient- and diameter-centered propensity score–matching of the two cohorts revealed no significant differences in histologic features regarding inflammation, fibrosis, or angiogenesis.
• **Take Home Message:** Histologic features do not differ in the aneurysmatic abdominal aortic wall of patients with abdominal aortic aneurysms vs patients with thoracoabdominal aneurysms.

The aorta is the body’s largest vessel, enabling a continuous blood flow to every major arterial branch from head to pelvis within every heartbeat by its elastic nature. Although the gross appearance comprising intima, media, and adventitia remains similar from the ascending aorta to the aortic bifurcation—the embryologic origin of this elastic type artery varies.^1^ Based on cellular ontology, vascular smooth muscle cells, the main cellular component of the aorta’s tunica media, arise from the second heart field (aortic bulb), the ectodermal cardiac neural crest (aortic arch), the somatic mesoderm (descending aorta to segment IV), and the splanchnic mesoderm (juxtarenal aorta and below).^2^^,^^3^

In nonhereditary conditions, aortic aneurysm formation, the pathologic enlargement of more than 1.5× age- and sex-adjusted baseline diameter is most frequently seen at the abdominal part (AAA), comprising more than 80% of idiopathic disease.^4^^,^^5^ However, more extended pathologies, such as thoracoabdominal aortic aneurysms (TAAAs), span from the proximal descending aorta to the iliac bifurcation.

In the past years, comparative studies have delineated histologic, expressional, and cellular functionality differences between hereditary aneurysm formations, such as Marfan syndrome, Loeys-Dietz syndrome, or bicuspid valve, as well as sole thoracic aneurysms, most often familial (ie, thoracic aortic aneurysm and dissection) and idiopathic disease at the respective localization.^1^^,^^6^, ^7^, ^8^, ^9^, ^10^ Here, the Society for Cardiovascular Pathology and the Association For European Cardiovascular Pathology have introduced a consensus document on nomenclature and diagnostic criteria.^11^ However, for the abdominal aorta, detailed pathohistologic investigations are scarce, specifically regarding the comparison of different disease entities.^12^

Our group has recently demonstrated that AAA wall samples differ significantly regarding inflammatory infiltrates, independent of aneurysm diameter, yet decreasing with age.^13^ Given the difference in embryologic origin and to better understand the histomorphology among heterogeneous aneurysm disease, this study aims to compare the pathohistology of aneurysmatic abdominal aortic samples from patients with AAAs and TAAAs.

## Methods

Additional information on all subsections can be found in the Supplementary Methods (online only).

### Patient identification

Patients with aneurysm were classified based on computed tomography angiography as AAA (abdominal aorta only: infra-/juxtarenal) or TAAA (all types according to the Crawford classification).^5^^,^^14^ Patients with known connective tissue diseases were not included (n = 2 in the database with >800 patients with aortic aneurysm). Abdominal aortic wall samples were collected from the anterior part of the circumference during open abdominal aortic repair (OAR). Indications for OAR in patients with AAAs were patient will, operator’s preference/expertise, or unsuitability for endovascular aortic repair. Indications for the very specific abdominal-only OAR in patients with TAAAs were rupture/symptomatic status of the abdominal part, pre-emptive abdominal repair for later endovascular treatment (ie, unsuitable iliac access), or other specific treatment strategies.

The study was performed in accordance with the declaration of Helsinki, and tissue sampling was approved by the local ethics committees of the Ethikkommission Klinikum Rechts der Isar: 2799/10. This specific study was additionally approved as part of the HistAAA study previously published (Ethikkommission Klinikum Rechts der Isar: 576/18S).^13^

### Patient and clinical data

Basic clinical data included is described in Table I.

**Table I:** Patient and aneurysm characteristics before and after matching

Characteristics	Available cohorts			4:1 Matched comparison		
	AAA	TAAA	*P*	AAA	TAAA	*P*
No. patients	272	24		96	24	
Male sex	231 (84.9)	17 (70.8)	.07	74 (77.1)	17 (70.8)	.52
Age, years	68.9 (63-75)	69.5 (58-79)	.97	70.1 (64-74)	69.5 (58-79)	.58
Rupture	43 (15.8)	8 (33.3)	**.03**	29 (30.2)	8 (33.3)	.77
D_max_, mm	62 (15.2)	70.1 (18.3)	**<.01**	68.1 (18)	70.1 (18.3)	.46
Comorbidities						
Hypertension	221 (81.3)	22 (91.7)	.2	86 (89.6)	22 (91.7)	.76
Diabetes	48 (17.6)	3 (12.5)	.52	11 (11.5)	3 (12.5)	.89
Hyperlipidemia	157 (57.7)	13 (54.2)	.74	49 (51)	13 (54.2)	.78
CAD	123 (45.2)	15 (10.9)	.11	62 (64.6)	15 (62.5)	.85
COPD	53 (19.5)	7 (29.2)	.26	27 (28.1)	7 (29.2)	.92
PAD	56 (20.6)	5 (20.8)	.98	23 (24)	5 (20.8)	.75
Smoking (overall)	206 (75.7)	17 (70.8)	.59	70 (72.9)	17 (70.8)	.84
Medication						
ASA/clopidogrel	173 (63.6)	15 (62.5)	.91	63 (65.6)	15 (62.5)	.77
ACE inhibitor	86 (31.6)	8 (33.3)	.86	32 (33.3)	8 (33.3)	1
Statin	135 (49.6)	12 (50)	.97	45 (46.9)	12 (50)	.78
Laboratory results						
CRP, mg/dL	2 (4.1)	3 (4.1)	.03	3.1 (5.4)	3 (4.1)	.23
Leukocyte, × 1000/μL	8.3 (3.1)	9.5 (4.3)	.21	8.8 (3.4)	9.5 (4.3)	.63
Thrombocyte × 1000/μL	213 (63)	262 (87)	**.02**	214 (68)	262 (87)	**.04**
Serum creatinine mg/dL	1.4 (1.3)	1.4 (1.1)	.55	1.8 (1.4)	1.4 (1.1)	.92

*AAA*, Abdominal aortic aneurysm; *ACE*, Angiotensin-converting enzyme; *ASA*, acetyl salecylic acid; *CAD*, coronary artery disease; *COPD*, chronic obstructive pulmonary disease; *CRP*, C-reactive protein; *D*_*max*_, maximum transverse centerline-based abdominal diameter; *PAD*, peripheral arterial disease; *TAAA*, thoracoabdominal aortic aneurysm.

Data are presented as number (%), mean (standard deviation), or mean (interquartile range).

Boldface *P* value indicates statistical significance (*P* < .05; χ^2^/*t*-test).

Items in italics were used for propensity score matching.

Laboratory results: CRP <0.5 mg/dL, leukocyte count <10.000/μL, thrombocytes count <450.000/μL; serum creatinine normal range, 0.8-1.1 mg/dL.

### Sample acquisition, preparation, and digitalization

Samples were collected and processed as previously described.^13^^,^^15^ Hematoxylin and eosin and Elastica van Gieson staining were performed according to standard protocols. Movat’s pentachrome staining was done according to the Verhöff protocol (Morphisto).

### Histologic analysis

All AAA/TAAA samples were analyzed by three pathologists according to the criteria published recently in detail, blinded to the AAA/TAAA groups.^13^ Briefly, the media was scored according to presence or absence of calcification, degree of inflammation, cellular composition of inflammatory infiltrates, presence of angiogenesis, and remaining elastic fibers vs full loss. Adventitial features were scored according to inflammatory properties and degree of fibrosis. Details on gradings are described in the Supplementary Methods (online only).

**Supplementary Fig 1:**
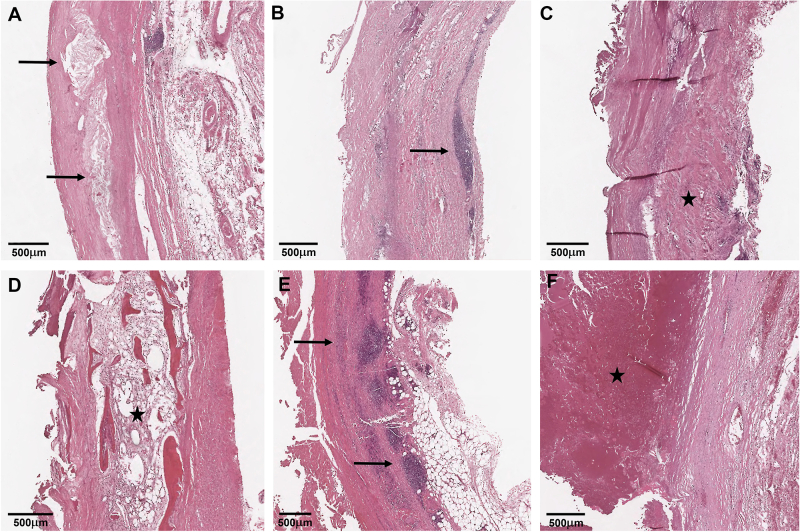


The type and degree of inflammation in the media and adventitia are highly significantly associated.^13^ Thus, for further analysis, the grade of inflammation was summarized for both layers. Similarly, the type of inflammation was summarized as acute (mixed infiltrate + granulocytes) or chronic (mononuclear cells + plasma cells) type (Supplementary Fig 1, online only).

### Statistics

Statistical analysis was performed using IBM SPSS for Windows, Version 30.0 (IBM Corp). To minimize potential bias due to unequal group distributions and to allow for a valid comparison of histologic features between patients with AAAs and TAAAs, a 4:1 nearest neighbor propensity score matching was performed (RStudio version 4.0.3 2024 MatchIt package; Posit Software, PBC). Matching was based on key patient- and aneurysm-specific characteristics to reduce confounding (see below in Results section). The quality of matching was evaluated by comparing standardized mean differences before and after matching. A standardized mean difference of <0.10 was considered indicative of adequate balance.

## Results

**Fig 1:**
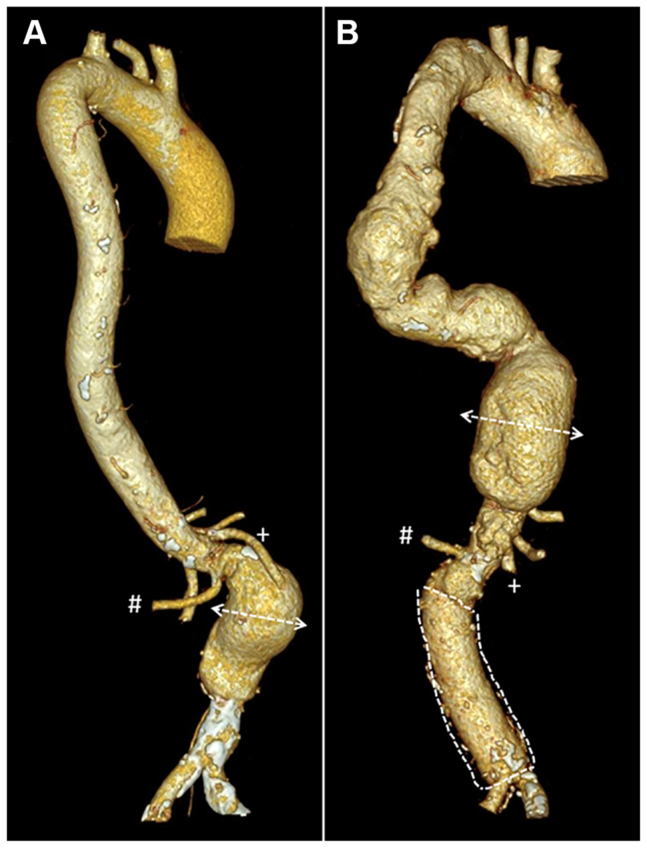
Three-dimensional computed tomography angiography reconstruction from representative patients (*MIP*, maximum intensity projection: thrombus not visible). **(A)** Dorsal view for a typical patient with juxtarenal AAA (male, 76 years old; *dotted arrow*, maximum axis corrected diameter 65 mm) before open repair (**#**, left renal artery; **+**, right renal artery). **(B)** Dorsal view of a patient with TAAA (female, 67 years old; *dotted arrow*, maximum axis corrected thoracic diameter 65 mm; previous maximum axis corrected abdominal diameter 60 mm) after infrarenal abdominal repair with a 28-mm Dacron tube graft (*dotted line*). Samples were collected from the anterior wall of the abdominal part of the aneurysm around the largest circumference in both cases.

**Fig 2:**
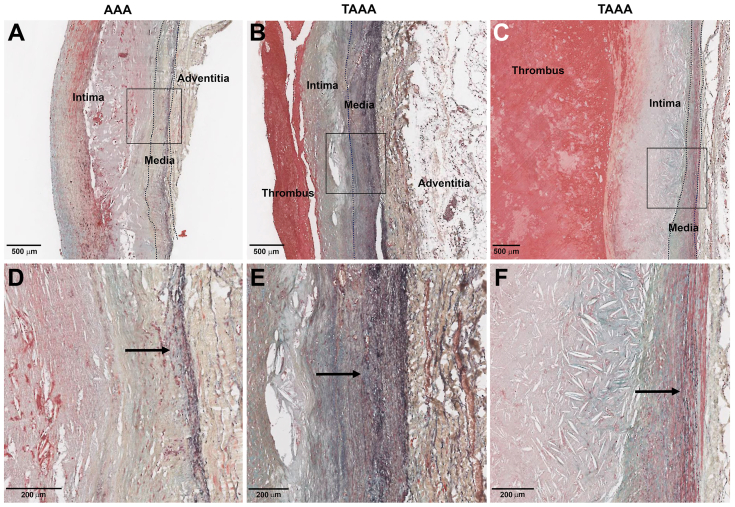
Abdominal aortic aneurysm (*AAA*) vs thoracoabdominal aortic aneurysm (*TAAA*) histologic comparison. **(A)** Aortic wall of patient with AAA with subtotal loss of elastic fibers of the media and fibrosis of the adventitia, Movat; **(B)** Aortic wall of patient with a TAAA, the elastic fibers of the media are slightly more preserved, with adherent thrombus, Movat; **(C)** Wall of patient with a TAAA, in this case with large adherent thrombus; **D-F,** Close-up of upper row: the *arrow* marks the remaining elastic fibers of the media, Movat.

Overall, 24 TAAA abdominal samples and the respective patient data were eligible for further analysis. These included 11 type II and 13 type III aneurysms (12 asymptomatic, 8 ruptured, 4 symptomatic). For comparison, AAA samples from 272 patients were available (Fig 1; Table I). Fig 2 provides an overview of AAA vs TAAA samples in Movat’s stain for gross depiction.

The TAAA and AAA cohorts showed significant differences regarding clinical presentation (AAA: 199 asymptomatic, 43 ruptures, 29 symptomatic; *P* = .03) and maximum centerline-based abdominal aortic diameter (AAA vs TAAA: 62 ± 15.2 vs 70.1 ± 18.3 mm; *P* < .01). Also, a higher proportion of female patients was found in the TAAA cohort (28.2% vs 15.1%; *P* = .07) (Table I).

To enable adequate statistical comparison of pathohistologic features between the two disease entities, a 4:1 cohort matching was performed for age, sex, clinical presentation, maximum diameter (abdominal/infrarenal part of TAAAs), comorbidities, smoking, and basic medication (thrombocyte inhibitors, angiotension-converting enzyme inhibitors, and statins) to “control” for patient and aneurysm characteristics. After matching, only a difference in absolute thrombocyte counts was seen, yet no patient showed out-of-range thrombocyte counts (Table I).

**Fig 3:**
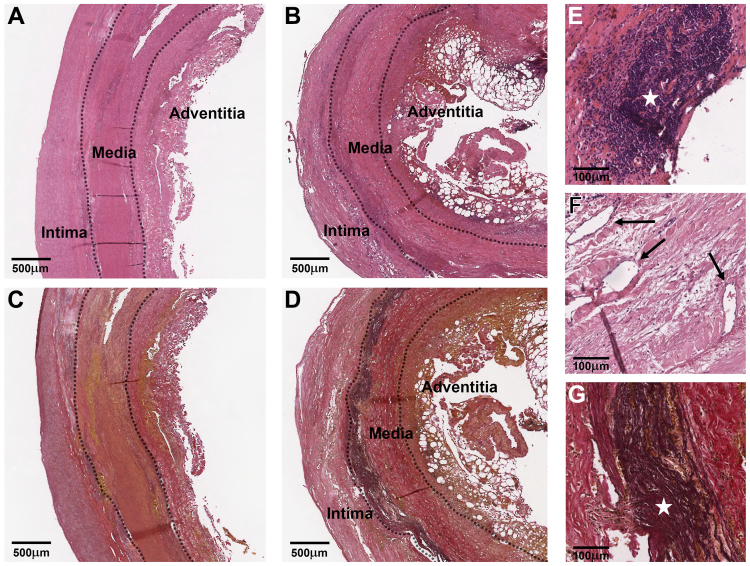
Abdominal aortic aneurysm (AAA) vs thoracoabdominal aortic aneurysm (TAAA) detailed histologic comparison. Aortic wall sample of patients with AAAs with subtotal loss of elastic fibers of the media and fibrosis of the adventitia shown in hematoxylin and eosin **(A)** and Elastica van Gieson **(C)** staining. Abdominal aortic wall sample of a patient with TAAA showing the elastic fibers of the media slightly more preserved in this very case upon hematoxylin and eosin **(B)** and Elastica van Gieson **(D)** staining. Closeup of an example of **(E)** mononuclear inflammatory infiltrate of the adventitia (∗); Closeup of an example of **(F)** neovascularization of the media (*arrows*); Closeup of an example of **(G)** the preserved yet crimpled elastic fibers from **(D)**.

Histologic analysis could be completed in every sample. The items addressed are shown exemplary in Fig 3. Here, no significant differences were observed regarding fibrosis, angiogenesis, or medial/adventitial inflammatory type and amount of infiltration (Table II; Supplementary Fig 1 [online only]). Of note, no differences in American Heart Association atherosclerosis classification of the samples were seen.

**Table II:** Comparison of histomorphology

Characteristics	4:1 matched comparison		
	AAA	TAAA	*P*
Patients	96	24	
Media			
Inflammation			
Degree			
0	54 (62.8)	14 (58.3)	0.38
1	30 (34.9)	8 (33.3)	
2	2 (2.3)	2 (8.3)	
3	0	0	
Type			
No	54 (62.8)	14 (58.3)	.34
Monocytes (C)	17 (19.8)	8 (33.3)	
Granulocytes (A)	–	–	
Plasma cells (C)	6 (7)	–	
Mix (A)	9 (10.5)	2 (8.3)	
Presence of angiogenesis	33 (37.5)	8 (33.3)	.71
Full elastic fiber loss	93 (96.8)	22 (91.7)	.25
Adventitia			
Inflammation			
Degree			
0	3 (3.3)	2 (8.7)	.73
1	57 (62.6)	14 (6.9)	
2	26 (28.6)	6 (26.1)	
3	5 (5.5)	1 (4.3)	
Type			
No	3 (3.3)	2 (8.7)	.62
Monocytes (C)	43 (47.3)	12 (52.2)	
Granulocytes (A)	1 (1.1)	–	
Plasma cells (C)	27 (29.7)	7 (3.4)	
Mix (A)	17 (18.7)	2 (8.7)	
Fibrosis			
No	–	–	.38
Mild	27 (29.7)	6 (26.1)	
Average	42 (46.2)	14 (60.9)	
Severe	22 (24.2)	3 (13)	
Summary			
Inflammation sum			
0	8 (8.3)	2 (8.3)	.07
1	35 (36.5)	12 (50)	
2	40 (41.7)	3 (12.5)	
3	10 (10.4)	5 (20.8)	
4	3 (3.1)	2 (8.3)	
Inflammation type			
0	8 (8.3)	2 (8.3)	.42
A	19 (19.8)	2 (8.3)	
C	69 (71.9)	20 (83.3)	
Atherosclerosis classification			
Class 5	9 (9.4)	2 (8.3)	.23
Class 6	82 (85.4)	19 (79.2)	
Class 8	5 (5.2)	3 (12.5)	

*A*, Acute; *AAA*, abdominal aortic aneurysm; *AHA*, American Heart Association; *C*, chronic; *TAAA*, thoracoabdominal aortic aneurysm.

Data are presented as absolute numbers and percentages.

Boldface *P* value indicates statistical significance (*P* < .05; χ^2^ test).

## Discussion

This study demonstrates that abdominal aortic wall sections from a well-matched cohort of patients with AAAs and TAAAs exhibit a similar pathomorphology in terms of type and degree of inflammation, presence of angiogenesis, and extent of fibrosis.

Basic scientific research on TAAA samples is rare due to the limited availability from open repair procedures. Despite good results from such invasive procedures in dedicated centers, today, patients with TAAAs receive primarily endovascular repair, except for patients with ascending aorta and aortic arch aneurysms.^10^^,^^14^^,^^16^^,^^17^ In addition, a vast part of TAAAs present as postdissection aneurysms and thus reflect a different clinical entity.

Although for thoracic aortic aneurysm, specifically hereditary forms, typical histologic patterns such as mucoid extracellular matrix accumulation might be different than the changes observed here, others, such as elastic fiber fragmentation and/or loss and laminar medial collapse are common signs of aneurysmal dilation.^11^ In specific conditions (ie, Marfan syndrome), heterogeneities, not only between thoracic or abdominal specimens, but also between connective tissue disease and “standard” patients, might be even more pronounced, most likely in coherence to different genetics and possibly a different importance of genetics overall.^6^^,^^17^^,^^18^ Yet, standardization of histologic features for AAA is lacking, and various groups have aimed to introduce such, the oldest dating back to 1994.^13^^,^^19^^,^^20^ Pucci et al have even suggested the addition of a cardiovascular pathologist to an aortic team.^21^

Although translational research on AAAs might not necessarily be transferable to TAAAs, the results presented here provide the first hint that, at least for the abdominal aortic part, patterns of inflammation and degenerative changes are comparable between the two cohorts. Our group was recently able to identify an age-related, but not a diameter-related, inverse association of inflammation, pointing towards more biological properties of the aneurysm wall in younger patients with AAAs as compared to rather “mechanical only” properties in elder patients.^13^ Possible nonsurgical aneurysm growth abrogation by different medications has been well-investigated for AAAs; yet, promising targets have not yet been successfully extracted.^22^ However, experimental studies have shown promising results in terms of multityrosine kinase inhibitors or metformin, for example, whereas clinical applications are still missing.^23^^,^^24^ In addition, imaging properties for specific characteristics of the aneurysm wall linked to possible rupture from AAA cohorts might be worthwhile investigating for TAAAs as well.^25^^,^^26^ However, diameter development is known to differ between AAAs and sole thoracic pathologies and might be different at specific parts of the aorta in TAAAs, with the abdominal part, for example, growing at a faster speed than the thoracic or vice versa— this is currently lacking sufficient data.^27^^,^^28^

Due to the limited number of available samples and the heterogeneity of patients and indications, this study can only be considered a strictly descriptive pilot investigation. Also, the specific site of sample acquisition from the abdominal parts of TAAAs only might not be representative for the entire aorta, given the unlike embryologic origin described above. Naturally, a type II error cannot be ruled out. All results are of a descriptive nature only. Interobserver bias for histologic evaluation must also be considered; yet, this is supposed to be minimal applying an objective deductive pattern of histologic features.

## Conclusions

Based on a limited number of samples comparing the very specific abdominal part of TAAA samples to AAA samples from a clinically 1:4 matched cohort, a similar pathomorphology is observed between both disease entities. Hence, aortic histomorphology should be considered in future translational research.

## Funding

Research was funded by the 10.13039/501100005971German Heart Foundation (Deutsche Herzstiftung: grant number F/46/18).

## Disclosures

None.

## Appendix (online only)

Supplementary docx 1
