# Supplementary material for: Histomorphologic characteristics of abdominal aortic aneurysm samples are similar in abdominal-only vs thoracoabdominal involvement
Source: JVS Vasc Sci. 2026 May 8;7:100424. doi: 10.1016/j.jvssci.2026.100424 (PMC13312072; doi:10.1016/j.jvssci.2026.100424)
Supplement: Supplementary docx 1 [file mmc1.docx]

**Supplementary patients, samples and Methods**

Patient identification

Samples and patient information were identified from the Munich Aortic biobank as described before.^1^ Aneurysm patients were classified according to their CT angiography based aneurysm morphometry as AAA or TAAA patients.^2,3^ Patient with known connective tissue diseases were not included (n = 2 in the database with > 800 patients with open repair). No post-dissection aneurysms were included. Samples were collected from the anterior part of the circumference during open abdominal aortic repair (OAR). Indications for OAR in AAA patients were patient will, operator’s preference/expertise or unsuitability for endovascular repair (EVAR). Indications for abdominal OAR in TAAA patients were rupture/symptomatic status of the abdominal part, pre-emptive abdominal repair for later endovascular treatment (i.e. unsuitable iliac access) or other specific treatment strategies. Collected samples were screened from 2005 – 2019.

All eligible patients were included consecutively in the biobank upon written and informed consent, where possible. For emergency patients, consent was acquired from relatives or the patient after initial recovery. Patient data was pseudonymized for biobanking and anonymized for further analysis. The study was performed in accordance with the declaration of Helsinki and tissue sampling was approved by the local ethics committees of the *Ethikkommission Klinikum rechts der Isar*: 2799/10. This specific study was approved as part of the HistAAA study previously published (*Ethikkommission Klinikum rechts der Isar*: 576/18S).^4^

Basic patient and clinical data

Basic clinical data included is described in Table I and in the Supplement section: age, sex (male/female), aneurysm state (symptomatic, ruptured, asymptomatic), maximum abdominal aortic diameter (maximum transverse diameter applying multiplane reconstructions allowing axis-corrected manual center-line based assessment) from 1-5 mm CT-angiographies 0-14 days prior to OAR (measurements performed by board-certified vascular surgeon at the abdominal location), AAA localization (abdominal including infra-, juxta-, pararenal; infrarenal = neck length ≥ 10mm) or TAAA type according to the Crawford classification, co-morbidities (hypertension, diabetes, hyperlipidemia, coronary artery disease CAD, chronic obstructive pulmonary disease COPD, peripheral artery disease (PAD), smoking (overall=current/ex/never), medication (anti-thrombocyte-aggregation, angiotensin-converting-enzyme inhibitor, statin) and laboratory results (C-reactive protein CRP, leukocyte/ thrombocyte count, serum creatinine).^5^

Sample acquisition, preparation and digitalization

After removal from the intraoperative situs, tissue was immediately rinsed in phosphate-buffered saline for transportation into the laboratory.^1^

Samples were then fixed in formalin (4% PFA) for 24 hours. If necessary, decalcification on EDTA basis (Entkalker soft SOLVAGREEN®, Carl ROTH, Karlsruhe, Germany) was performed for 2-7 days. Afterwards specimens were prepared for paraffin embedding in standard size (40 x 28 x 6.8 mm) POM histology cassettes (Kartell, Noviglio, Italy). Sections of parafﬁn-embedded samples (2 µm for classic samples) were mounted on glass slides (Menzel SuperFrost, 76 x 26 x 1 mm, Fisher Scientific, Schwerte, Germany).

Hematoxylin-eosin (HE) (ethanolic eosin Y solution, Mayer’s acidic hemalum solution, Waldeck, Münster, Germany) as well as elastica van Gieson (EvG) (picrofuchsin solution after Romeis 16th edition, Weigert’s solution I after Romeis 15th edition) stainings were accomplished according to the manufacturer’s protocol. Slides were covered using Pertex (Histolab products, Askim, Sweden) as mounting medium and glass coverslips (24 x 50 mm, Engelbrecht, Edermünde, Germany).

Slides (including immunohistochemistry) were then scanned with Aperio AT2 (Leica, Wetzlar, Germany), and pictures were taken with the Aperio ImageScope software (Leica). Scanned slides were analyzed and prepared for composite figures using QuPath-0.3.2 open-source software.^6^

Histologic Analysis

All AAA samples were analyzed by three pathologists as published previously according to the criteria published recently in detail.^4^ TAAA samples were analyzed by one of the three pathologists for this specific study.

*Media*: The media was scored according to presence or absence of calcification (0 = absence, 1 = presence), degree of inflammation (0 = no inflammation; 1 = low, 2 = intermediate, 3 = high degree of inflammation), composition of inflammatory infiltrate (1 = mainly composed of mononuclear cells, 2 = granulocytes, 3 = plasma cells or 4 = mixed infiltrate), presence of neoangiogenesis (= visible nests of neovessels) (0 = absence, 1 = presence) and the remaining vs. full loss of elastic fibers.

*Adventitia*: Adventitial features were scored according to degree of inflammation (s. above), composition of inflammatory infiltrate (s. above) and degree of fibrosis (0 = no fibrosis, 1 = low, 2 = intermediate, 3 = high degree of fibrosis).

Degrees of inflammation and fibrosis were defined as follows:

*Inflammation*: 0 = no or only singular inflammatory cells; 1 = localized small infiltrates; 2 = localized and diffuse infiltrates; 3 = diffuse dense infiltrates.

*Fibrosis*: 0 = no proliferation of collagenous fibers; 1 = up to a third of visible adventitia with collagenous fiber proliferation; 2 = up to a half of visible adventitia with collagenous fiber proliferation; 3 = more than half of visible adventitia with collagenous fiber proliferation.

The type and degree of inflammation in the media and adventitia were highly significantly associated. Specifically, adventitial low inflammatory grade was likely to have a low medial inflammatory grade.^4^ Thus, for further analysis, the grade of inflammation was summarized for both layers. Similarly, the type of inflammation was summarized as acute (mixed infiltrate + granulocytes) or chronic (mononuclear cells + plasma cells) type. The updated American Heart Association (AHA) classification for atherosclerotic lesions was applied to the samples as suggested by the *Consensus statement on surgical pathology of the aorta from the Society for Cardiovascular Pathology and the Association for European Cardiovascular Pathology* .^7,8^

Statistics

Statistical analysis was performed using IBM SPSS for Windows, Version 30.0 (IBM Corp., Armonk, NY). All clinical characteristics were grouped to build categorical or nominal variables. Dichotomous variables were recorded as absolute frequencies (number of cases) and relative frequencies (percentages). Continuous data are presented as mean and standard deviation, non-symmetrical with median and interquartile range (IQR). Pearson’s chi-squared or Fisher’s exact test was used to analyze categorical variables. Differences between means were tested with t-test or Mann-Whitney-U-test. P-values < 0.05 were considered statistically significant. To minimize potential bias due to unequal group distributions and to allow for a valid comparison of histological features between AAA and TAAA patients, a **4:1 propensity score matching (PSM)** was performed. Matching was conducted using RStudio version 4.0.3 2024 (Posit Software, PBC, Boston, MA) with the **MatchIt** package. The propensity score was estimated algorithmically based on the following covariates: age, sex, clinical presentation (asymptomatic/ /ruptured), maximum aortic diameter, comorbidities (hypertension, diabetes mellitus, hyperlipidemia, coronary artery disease, COPD, peripheral artery disease), smoking status and baseline medication (antiplatelet agents, ACE inhibitors, statins). Matching was performed using a **nearest-neighbor algorithm. The quality of matching was evaluated by comparing standardized mean differences (SMD) before and after matching. An SMD of < 0.10 was considered indicative of adequate balance.**

Data visualization was performed with SPSS, Microsoft Excel and RStudio the latter along with extension packages ggplot2 (visualization).

**Supplementary references**

1. Pelisek J, Hegenloh R, Bauer S*, et al.* Biobanking: Objectives, Requirements, and Future Challenges-Experiences from the Munich Vascular Biobank. *J Clin Med* 2019;**8**. doi: 10.3390/jcm8020251

2. Wanhainen A, Van Herzeele I, Bastos Goncalves F*, et al.* Editor's Choice -- European Society for Vascular Surgery (ESVS) 2024 Clinical Practice Guidelines on the Management of Abdominal Aorto-Iliac Artery Aneurysms. *Eur J Vasc Endovasc Surg* 2024;**67**:192-331. doi: 10.1016/j.ejvs.2023.11.002

3. Riambau V, Bockler D, Brunkwall J*, et al.* Editor's Choice - Management of Descending Thoracic Aorta Diseases: Clinical Practice Guidelines of the European Society for Vascular Surgery (ESVS). *Eur J Vasc Endovasc Surg* 2017;**53**:4-52. doi: 10.1016/j.ejvs.2016.06.005

4. Nackenhorst MC, Menges F, Bohmann B*, et al.* Abdominal aortic aneurysm histomorphology shows different inflammatory aspects among patients and is not associated with classic risk factors - the HistAAA study. *Cardiovasc Res* 2025. doi: 10.1093/cvr/cvaf071

5. Wanhainen A, Verzini F, Van Herzeele I*, et al.* Editor's Choice - European Society for Vascular Surgery (ESVS) 2019 Clinical Practice Guidelines on the Management of Abdominal Aorto-iliac Artery Aneurysms. *Eur J Vasc Endovasc Surg* 2019;**57**:8-93. doi: 10.1016/j.ejvs.2018.09.020

6. Bankhead P, Loughrey MB, Fernandez JA*, et al.* QuPath: Open source software for digital pathology image analysis. *Sci Rep* 2017;**7**:16878. doi: 10.1038/s41598-017-17204-5

7. Stary HC. Natural history and histological classification of atherosclerotic lesions: an update. *Arterioscler Thromb Vasc Biol* 2000;**20**:1177-1178. doi: 10.1161/01.atv.20.5.1177

8. Halushka MK, Angelini A, Bartoloni G*, et al.* Consensus statement on surgical pathology of the aorta from the Society for Cardiovascular Pathology and the Association For European Cardiovascular Pathology: II. Noninflammatory degenerative diseases - nomenclature and diagnostic criteria. *Cardiovasc Pathol* 2016;**25**:247-257. doi: 10.1016/j.carpath.2016.03.002

**Supplementary Figures and Figure legends**

**
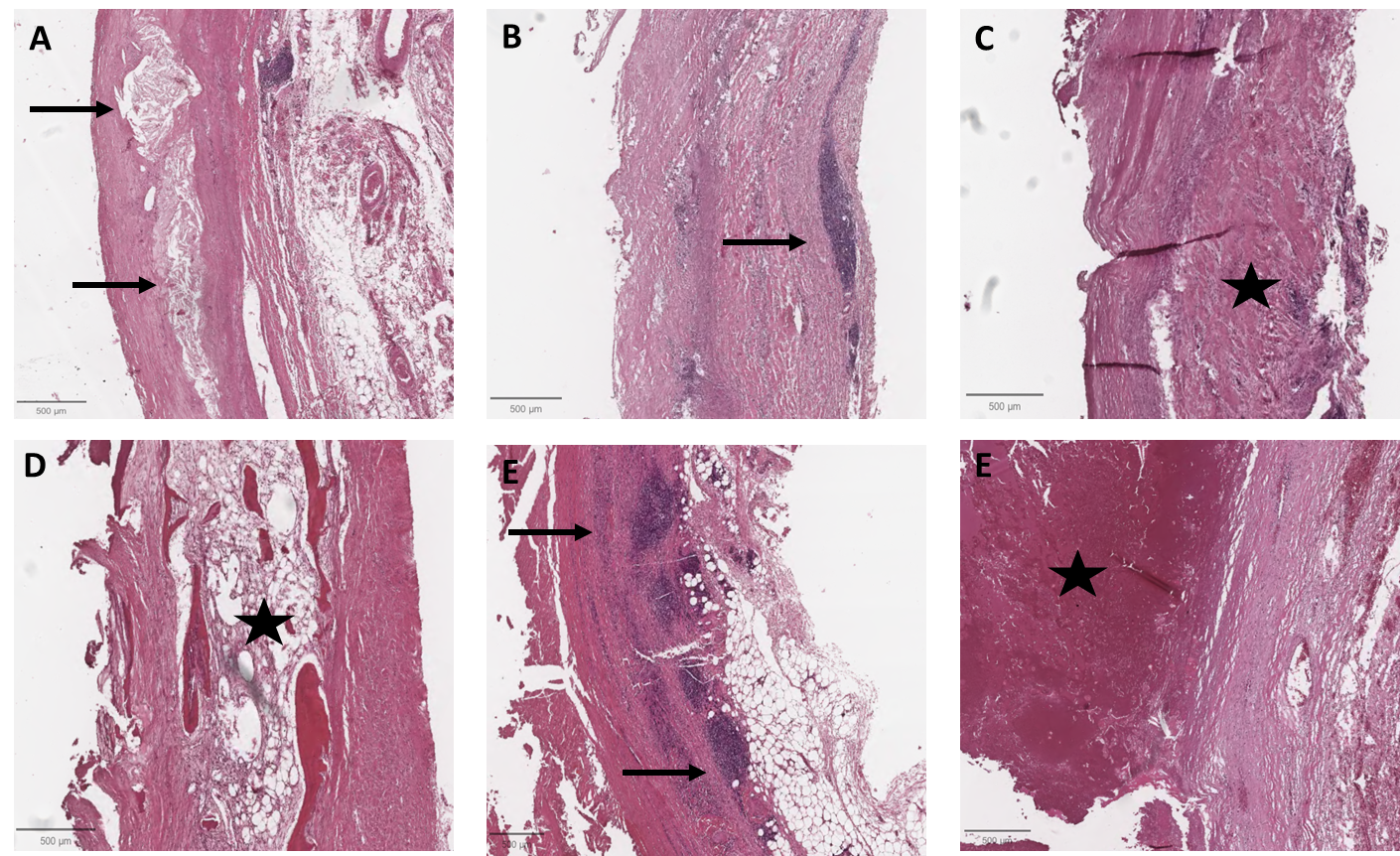
**

**Supplementary Figure 1. Range of histomorphologic features. (A)** and **(D)**: Intimal changes: **(A)** Atherosclerosis with cellular debris and cholesterol crystals in the intima (arrows); **(D)** metaplastic ossification (star); **(B)** and **(E)**: inflammatory changes: **(B)** adventitial mononuclear inflammation; **(E)** mononuclear inflammation affecting both adventitia and media (arrows); **(C)** Extensive fibrosis of the adventitia (star); **(F)** large thrombus (star)
